# Development of a novel *nannochloropsis* strain with enhanced violaxanthin yield for large‐scale production

**DOI:** 10.1186/s12934-021-01535-0

**Published:** 2021-02-15

**Authors:** Su-Bin Park, Jin-Ho Yun, Ae Jin Ryu, Joohyun Yun, Ji Won Kim, Sujin Lee, Saehae Choi, Dae-Hyun Cho, Dong-Yun Choi, Yong Jae Lee, Hee-Sik Kim

**Affiliations:** 1 Cell Factory Research Center, Korea Research Institute of Bioscience and Biotechnology (KRIBB), 34141 Daejeon, Republic of Korea; 2 Major of Environmental Biotechnology, KRIBB School of Biotechnology, Korea University of Science and Technology (UST), 34113 Daejeon, Republic of Korea; 3 Osong Medical Innovation Foundation, 28160 Chungbuk, Republic of Korea

**Keywords:** Violaxanthin, Microalgae, *Nannochloropsis oceanica*, Random mutagenesis, Gamma‐ray irradiation, Transcriptome

## Abstract

**Background:**

*Nannochloropsis* is a marine microalga that has been extensively studied. The major carotenoid produced by this group of microalgae is violaxanthin, which exhibits anti-inflammatory, anti-photoaging, and antiproliferative activities. Therefore, it has a wide range of potential applications. However, large-scale production of this pigment has not been much studied, thereby limiting its industrial application.

**Results:**

To develop a novel strain producing high amount of violaxanthin, various *Nannochloropsis* species were isolated from seawater samples and their violaxanthin production potential were compared. Of the strains tested, *N. oceanica* WS-1 exhibited the highest violaxanthin productivity; to further enhance the violaxanthin yield of WS-1, we performed gamma-ray-mediated random mutagenesis followed by colorimetric screening. As a result, Mutant M1 was selected because of its significant higher violaxanthin content and biomass productivity than WS-1 (5.21 ± 0.33 mg g^− 1^ and 0.2101 g L^− 1^ d^− 1^, respectively). Subsequently, we employed a 10 L-scale bioreactor to confirm the large-scale production potential of M1, and the results indicated a 43.54 % increase in violaxanthin production compared with WS-1. In addition, comparative transcriptomic analysis performed under normal light condition identified possible mechanisms associated with remediating photo-inhibitory damage and other key responses in M1, which seemed to at least partially explain enhanced violaxanthin content and delayed growth.

**Conclusions:**

*Nannochloropsis oceanica* mutant (M1) with enhanced violaxanthin content was developed and its physiological characteristics were investigated. In addition, enhanced production of violaxanthin was demonstrated in the large-scale cultivation. Key transcriptomic responses that are seemingly associated with different physiological responses of M1 were elucidated under normal light condition, the details of which would guide ongoing efforts to further maximize the industrial potential of violaxanthin producing strains.

## Background

*Nannochloropsis* is an oleaginous phytoplankton commonly found in coastal areas. Due to their high lipid content [1], several species in this genus have been investigated as a promising feedstock for the production of biodiesel [2, 3]. They have also been studied as a source of high-value fatty acids, such as eicosapentaenoic acid (EPA) [4, 5]. Furthermore, *Nannochloropsis* has been identified as one of the predominant industrial algal crops due to their rapid growth rate, recoverable high-value products, and recent reports of successful genetic transformation [6–11].

As the major R&D efforts in algal biotechnology were shifted towards utilizing less explored non-lipid portion of algal biomass based on the conceptualization of algal biorefinery and its apparent economic advantages, there has been a close attention to the diversification of algae-derived products beyond biofuels [12, 13]. In particular, carotenoid pigments derived from microalgae exhibit substantial nutraceutical and pharmaceutic values, and they have also been utilized as a key functional ingredient in the field of cosmetics [13–16]. These organic pigments are accessory pigments that are produced by photosynthetic organisms; and they are known to play an important role in remediating excess oxidative stress and absorbing the wavelengths of light that are not primarily utilized by chlorophylls [17–19].

Previous studies have demonstrated violaxanthin as a major carotenoid in *Nannochloropsis* [20, 21]. It has been reported that violaxanthin exhibits anti-inflammatory activity in macrophage cells along with antiproliferative activity against human cancer cell lines. [22, 23]. Furthermore, violaxanthin appeared as an excellent radical scavenger, and showed strong antioxidative capacity, thereby inhibiting hemolysis induced by H_2_O_2_ and lipid peroxidation [24]. Recent studies have also demonstrated the protective effect of violaxanthin against ultraviolet-B-induced skin damage (e.g., wrinkles), justifying the cosmetic applications of this pigment [25]. However, although various industrial sectors have discovered effective applications of violaxanthin, it has to be noted that the yield of violaxanthin is crucial in determining its commercial feasibility. To date, several efforts have been made in terms of modulating cultivation conditions to enhance the productivity of violaxanthin from *Nannochloropsis*. For instance, Forján Lozano et al. [26] demonstrated that the production of violaxanthin in *Nannochloropsis gaditana* could be enhanced in sulphate- or phosphate-limited conditions. In addition, outdoor cultivation of *N. gaditana* under mixotrophy was determined to lead to an increase in total carotenoid including violaxanthin compared with those grown under phototrophic condition [27]. However, optimizing different culture conditions would need to be combined with the development of key industrial strains to maximize the commercial potential of algae-based bioproducts and break into the current market [28].

Importantly, random mutagenesis can serve as an effective strategy to develop algal strains with desired industrial properties. For instance, Shin et al. [29] reported that ethyl methanesulfonate (EMS)-based mutagenesis resulted in the truncation of antennae pigment, which in turn substantially increased the photosynthetic efficiency of *Chlorella* sp. and a 44.5 % increment in biomass productivity compared with that of the wild type. Similarly, another study reported a random mutant of *Phaeodactylum tricornutum* obtained from adaptive laboratory evolution and noted that this mutant displayed an approximately two-fold higher fucoxanthin production and growth rate than the progenitor [30]. In addition, given that an increased yield of specific carotenoids would lead to a distinctive change in the color of algal colonies, the selection of candidate mutants following the random mutagenesis is likely to be highly effective, further justifying the deployment of random mutagenesis for developing high violaxanthin producing mutants.

In this study, we aimed to develop a mutant *Nannochloropsis* strain with high violaxanthin production potential based on gamma (γ)-ray mutagenesis. Multiple species of *Nannochloropsis* were first isolated from samples obtained from the west coast of Republic of Korea. Violaxanthin productivity in all isolates were first evaluated; a selected wild type with the highest violaxanthin content was then subjected to γ-ray irradiation to induce random mutagenesis. Subsequently, algal cells treated with γ-ray were spread onto agar plates, and yellow-colored colonies were screened. Following a series of subculturing, a mutant strain with improved violaxanthin content and biomass productivity was selected, and the yield of violaxanthin in this mutant was further compared with the progenitor strain under different conditions, including a large-scale bioreactor cultivation. In addition, comparative transcriptomic analysis of a selected mutant and the progenitor strains were performed to elucidate transcription-level responses under normal light condition that are seemingly associated with the enhanced violaxanthin production and other physiological changes in the mutant. Collectively, the results suggested our mutant as a reliable industrial strain for the production of violaxanthin, while further investigations seemed to be necessary to understand detailed molecular mechanisms associated with physiological responses of the mutant under different operational conditions.

## Results and discussion

### Isolation of *Nannochloropsis* strains and comparison of violaxanthin production

In general, violaxanthin is the major carotenoid produced by *Nannochloropsis*. Therefore, the violaxanthin content of *Nannochloropsis* is much higher than that of other green microalgae [21]. However, the violaxanthin content may vary among different *Nannochloropsis* species. Therefore, in this study, we tried to isolate the strain with the highest violaxanthin yield. For the isolation of different candidate strains, seawater samples from Korean west coast was spread onto F/2 agar plates, and those were incubated until colony growth was confirmed. Some of the green colonies were isolated and analyzed using 18S rDNA PCR followed by sequencing. As a result, we identified 6 different *Nannochloropsis* strains, which were classified based on 18S rDNA homology, and these strains were named in a numeric order form WS-1 to WS-6 (Table 1). Thereafter, these strains were cultivated in flasks to compare biomass productivity and violaxanthin content based on the samples collected on the last day of cultivation. Among the six strains, WS-1 exhibited highest biomass productivity of 0.30 g L^− 1^ d^− 1^, while WS-6 showed the lowest biomass productivity of 0.03 g L^− 1^ d^− 1^. Similarly, violaxanthin content of WS-1 was the highest as 3.93 mg g^− 1^ and that of WS-6 was the lowest as 0.21 mg g^− 1^ (Table 1). Although these results may indicate that violaxanthin content is proportional to the cellular growth, this was not the case for the other strains. For instance, WS-4 showed about 3 times higher biomass productivity than that of WS-3; however, the violaxanthin content of WS-4 was about half of WS-3. Finally, violaxanthin productivity was calculated by multiplying biomass productivity with violaxanthin content and was compared among the strains. As expected, violaxanthin productivity was highest in WS-1 (1.1615 mg L^− 1^ d^− 1^) and lowest in WS-6 (0.0056 mg L^− 1^ d^− 1^), respectively, which corresponded to a 207-fold increase in the violaxanthin productivity. Therefore, *Nannochloropsis oceanica* WS-1 was selected as a host for the subsequent experiments. In addition, the production of violaxanthin from this strain was validated again by analyzing a spiked sample consisting of algal pigment extract and violaxanthin standard. The results clearly indicated a single peak with the same retention time as the violaxanthin standard in the spiked sample, ensuring the presence of the target carotenoid pigment (Additional file 1: Fig. S1).

**Table 1:** Identification of *Nannochloropsis* species and comparative assesment of biomass and violaxanthin productivity

Strain	Closest species (% Identity of 18S rDNA)	Collection site (South Korea)	Latitude/Longitude	Biomass productivity (g L^− 1^ d^− 1^)	Violaxanthin content (mg g^− 1^)	Violaxanthin productivity (mg L^− 1^ d^− 1^)
WS-1	*N. oceanica* (99 %)	Gungni Port, Hongseong-gun,	36º35′30″N/126º27′20″E	0.2956	3.9298	1.1615
WS-2	*N. salina* (99 %)	Samgil Port, Seosan-si,	37º00′10″N/126º27′10″E	0.2372	3.7000	0.8777
WS-3	*N. oceanica* (99 %)	Doksan Beach, Boryung-si,	36º13′12″N/126º31′35″E	0.0739	2.0798	0.1537
WS-4	*N. oceanica* (100 %)	Janghang Beach, Seocheon-gun,	36º00′55″N/126º39′42″E	0.2139	1.1514	0.2463
WS-5	*N. gaditana* (100 %)	Janghang Beach, Seocheon-gun,	36º00′55″N/126º39′42″E	0.2722	3.4932	0.9509
WS-6	*N. gaditana* (98 %)	Doksan Beach, Boryung-si,	36º13′12″N/126º31′35″E	0.0272	0.2068	0.0056

### Random mutagenesis and mutant screening

Gamma-ray-mediated random mutagenesis was performed to generate *N. oceanica* WS-1 mutants. The dose of gamma-ray irradiation was modulated in the range of 100 to 1000 Gy. The viability of irradiation-treated cells was determined by counting the number of colonies. The results indicated that some of the cells survived gamma-ray irradiation as high as 500 Gy; however, no colonies were observed at 700 and 1000 Gy (Additional file 1: Table S1). Therefore, cells treated with 500 Gy of gamma-ray, the maximum irradiation dose for obtaining a viable cell, were used in mutant screening. In addition, since violaxanthin is orange-colored pigment, yellowish or pale colonies were chosen for random mutant selection.

**Fig. 1:**
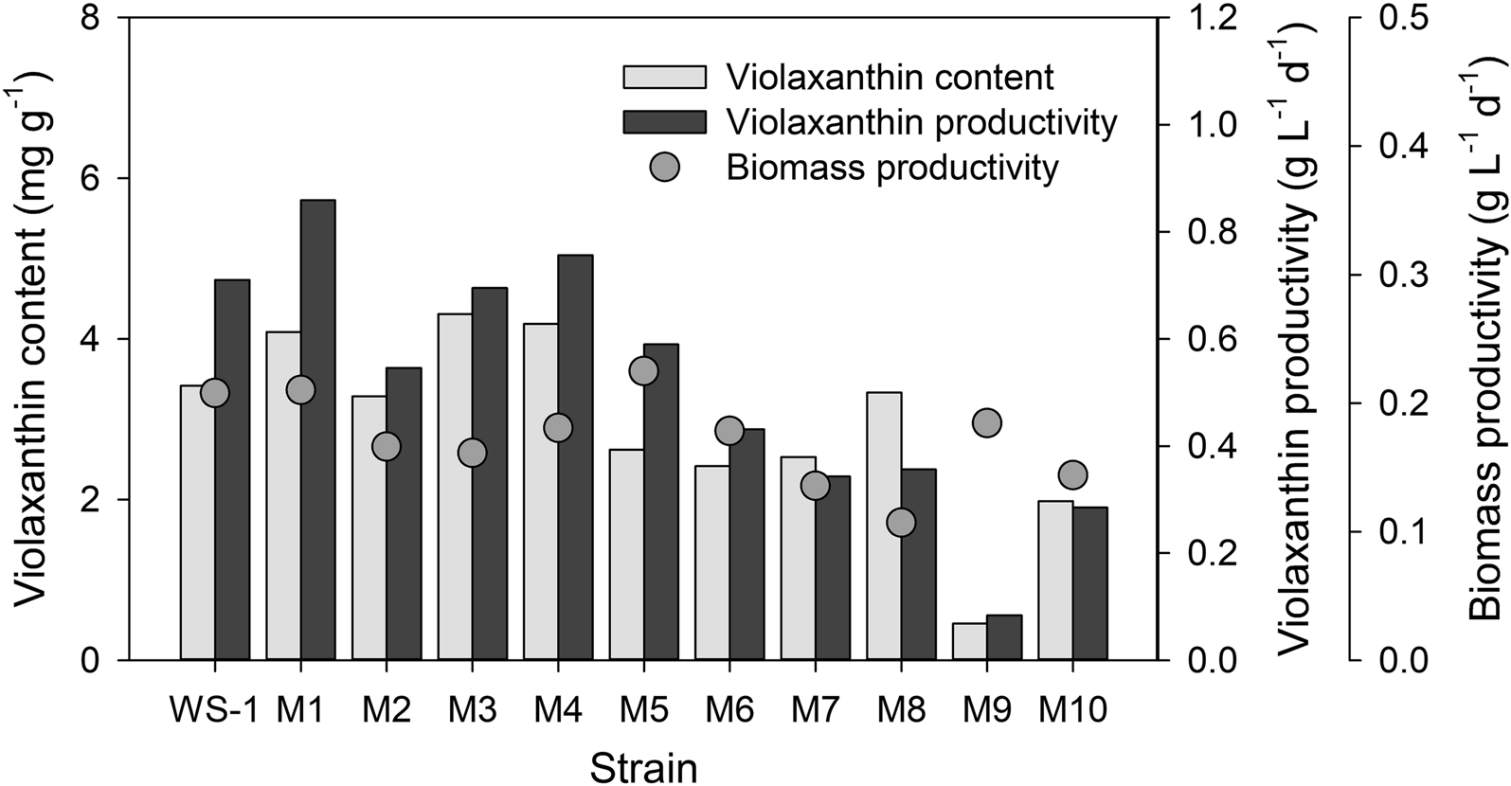
Comparison of biomass and violaxanthin productivity for the mutant screening. Grey bars and dots indicate violaxanthin content (mg g^− 1^), violaxanthin productivity (mg L^− 1^ d^− 1^) and biomass productivity (g L^− 1^ d^− 1^), respectively

To isolate the mutant with stable growth, the selected colonies were subjected to continuous subculture: 93 colonies were first inoculated individually and strains with poor growth were then excluded after repeating subculturing for 5 times every 15–20 days. 10 stable mutants were finally obtained, and biomass productivity and violaxanthin productivity of these strains were compared with those of *N. oceanica* WS-1. The results indicated that the biomass productivities of the mutant strains, M1 and M5 were respectively 0.2101 g L^− 1^ d^− 1^ and 0.2249 g L^− 1^ d^− 1^, which were higher than that of WS-1 (0.2078 g L^− 1^ d^− 1^) (Fig. 1 and Additional file 1: Table S2). On the other hand, the rest of the mutants exhibited the biomass productivity lower than 0.2 g L^− 1^ d^− 1^. Notably, violaxanthin content values of M1, M3 and M4 were higher than that of WS-1. Of these mutants, a comparison of violaxanthin productivity indicated M1 as the mutant with the highest violaxanthin productivity (i.e., 0.8589 mg L^− 1^ d^− 1^), while the violaxanthin productivity values of WS-1, M3, M4 were 0.7101 mg L^− 1^ d^− 1^, 0.6951 mg L^− 1^ d^− 1^, and 0.7563 mg L^− 1^ d^− 1^, respectively (Fig. 1 and Additional file 1: Table S2). In addition, it should be noted that the observed biomass productivity of M5 was higher than M1 and WS-1; however, violaxanthin content was much lower in M5 than these two strains. Compared with the progenitor WS-1, the highest violaxanthin productivity observed in M1 corresponded to a 21 % increase in violaxanthin productivity (Fig. 1 and Additional file 1: Table S2). On the contrary, the rest of the mutants excluding M4 and M5 exhibited substantially lower biomass productivity and violaxanthin content than WS-1. Based on these results, *N. oceanica* M1 was selected as a promising strain for the industrial production of violaxanthin, and its performance was further compared with that of WS-1 in the sections below.

### Effect of light intensity on growth and violaxanthin content

In general, cell growth and carotenoid productivity are closely related to light intensity. Several studies have already performed to optimize the light condition for microalgae cultivation [31–34]. However, the optimal light condition strongly depends on various factors, such as strain, cultivation scale, type of bioreactor, and cell density. In this section, the effect of light intensity on the yield of violaxanthin was investigated to determine the possibility of additional enhancement of the target carotenoid in two strains of interest (i.e., WS-1 and M1) under different light treatments. Specifically, 50 µmol photon m^− 2^ s^− 1^ of light was used as normal condition following the study of Spolaore et al. [31], because of its technical similarities with our study, including the deployment of side light irradiation and 24:0 LD cycle.

**Fig. 2:**
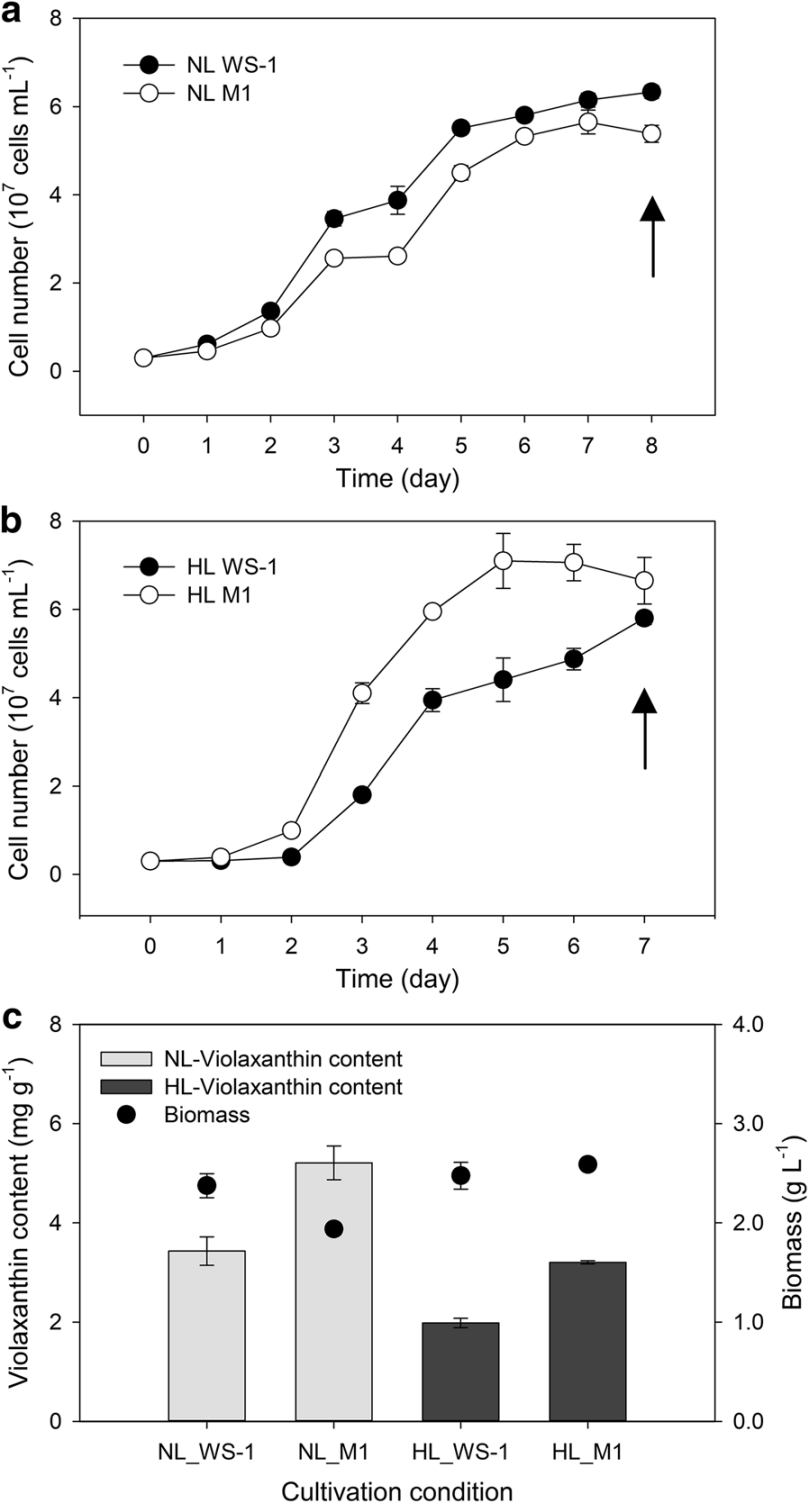
Results of flask cultivations. Growth curve of *N. oceanica* WS-1 and M1 under the **a** normal and **b** high light conditions. Closed and open circles indicate WS-1 and M1, respectively. **c** Violaxanthin contents and biomass production yield of *N. oceanica* WS-1 and M1 under different light conditions. Light and dark grey bars indicate violaxanthin contents (mg g^− 1^) under normal and high light conditions, respectively. Dots indicate biomass production yields (g L^− 1^). Arrows in **a** and **b** indicate time points at which sampling was performed to obtain data for **c**

To examine the effect of light intensity, we compared cell growth under the normal light (NL, 50 µmol photon m^− 2^ s^− 1^) and high light (HL, 150 µmol photon m^− 2^ s^− 1^) conditions. In case of WS-1, the cell density reached around 6 × 10^7^ cells mL^− 1^ after 7 days of cultivation under both conditions (Fig. 2a, b). In contrast, we observed faster growth in case of M1 under HL condition. In particular, the maximum cell density (7.10 × 10^7^ cells mL^− 1^) was observed only after 5 days (Fig. 2a, b) under HL, whereas 7 days of cultivation was required for M1 to reach the maximum cell density (5.65 × 10^7^ cells mL^− 1^) under the NL condition. In principle, optimal light intensity for efficient photosynthesis depends on the antenna size of the strain, and one of the factors influencing the antenna size is the composition of light-harvesting (antenna) pigments, including carotenoids [35, 36]. Given that an increase in light intensity did not have a substantial influence on biomass productivity in WS-1, a contrasting increment in the biomass productivity of M1 under HL condition denotes that the change of pigment composition in the mutant likely increased its light dependency, which could be associated with a shift in antenna size [37].

Subsequent comparison of violaxanthin content indicated a higher violaxanthin content in M1 than in WS-1 under both conditions (Fig. 2c). Generally speaking, HL is not a preferred condition for violaxanthin production and both strains exhibited a higher production of violaxanthin under NL than under HL condition (Fig. 2c). It should be also noted that the violaxanthin content of M1 was increased during a series of subculture: 4.0874 mg g^− 1^ of violaxanthin content was observed during the initial mutant screening period, whereas 5.21 ± 0.34 mg g^− 1^ of violaxanthin content (corresponding to the violaxanthin productivity of 10.08 ± 0.66 mg L^− 1^) was observed following a stabilization of this mutant with successive subculturing (Fig. 2c). Compared with other microbes that were studied for violaxanthin production, these results indicated one of the highest violaxanthin content reported. For example, *Muriellopsis* sp. was reported to contain 7.3 ± 0.3 mg of the violaxanthin per liter of culture solution, which corresponded to 1.359 mg of violaxanthin per gram of dried cell weight [38]. In addition, *Eustigmatos* cf. *Polyphem* was studied for various culture conditions for violaxanthin production, but it only contained about 0.4 % of the violaxanthin per dried cell weight [39]. Although a yeast strain transformed with carotenogenic genes from *Haematococcus lacustris* achieved a maximum violaxanthin production of 7.3 mg per gram of dried cell weight [40], another study reported a mutant of *Chlorella vulgaris* obtained following chemical mutagenesis and reported a violaxanthin content of 3.70 ± 0.45 mg per gram of dried cell weight, which was substantially lower than that of M1 [41].

### Large-scale cultivation for violaxanthin production

**Fig. 3:**
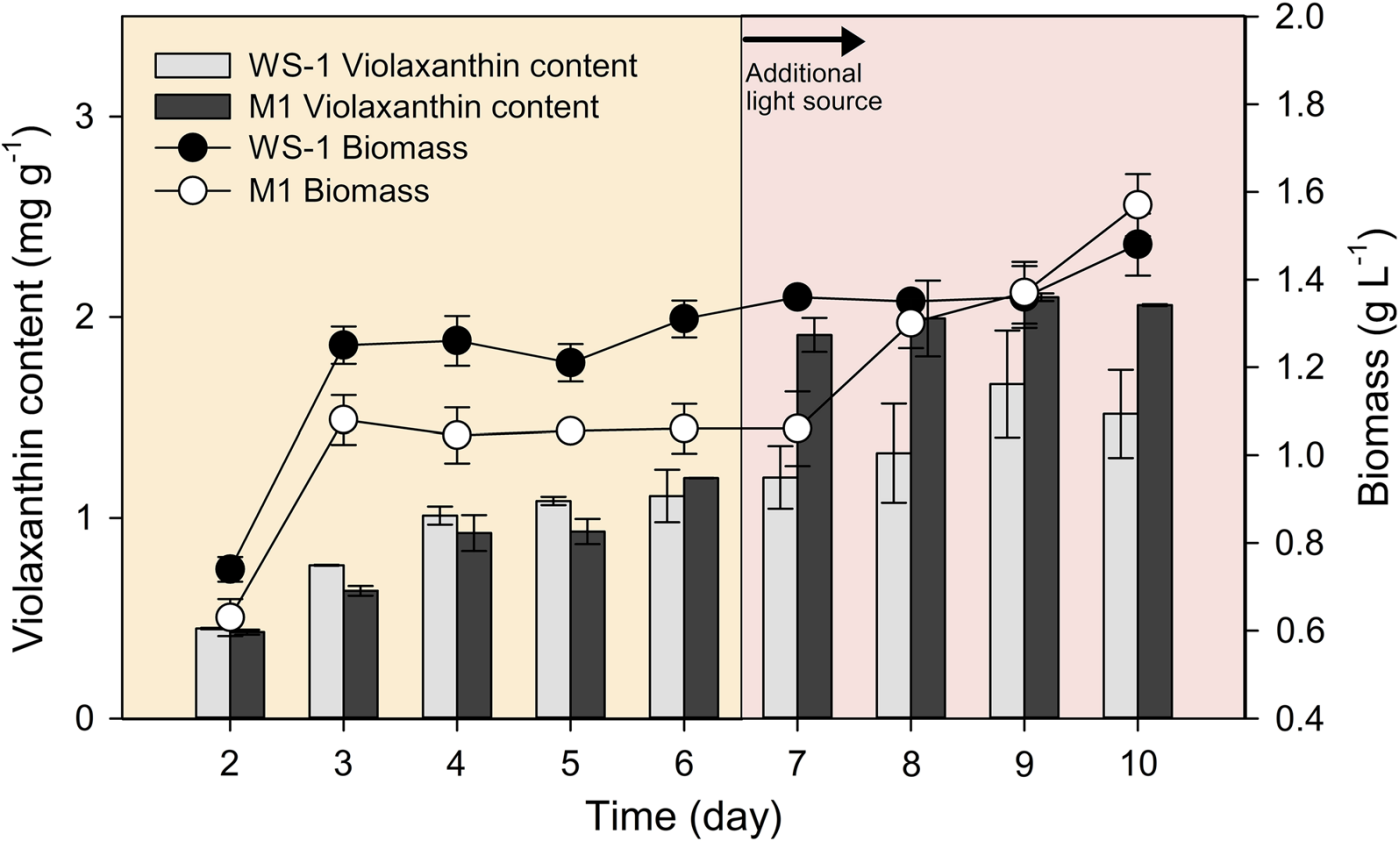
Growth curves and violaxanthin contents of large-scale cultivations. Closed and open circles indicate growth profiles of *N. oceanica* WS-1 and M1, respectively. Light and dark grey bars indicate violaxanthin contents (mg g^− 1^) of WS-1 and M1, respectively

To further evaluate the biomass and violaxanthin productivity in large-scale, cell cultivation was also carried out in 10 L bioreactor. In large-scale cultivation, light transmission efficiency is expected to diminish as the cell concentration increases (shading effect), which would require an increased light intensity to facilitate cellular growth [42, 43]. Therefore, an additional light source was supplemented on Day 6 to compensate the shading effect. During the entire cultivation period, biomass accumulation was monitored daily and the violaxanthin content was measured from Day 2. The results indicated the exponential growth of each strain until Day 3; however, both M1 and WS-1 did not exhibit significant biomass accumulation until Day 5. With the supplementation of additional light source, a rapid growth was observed in M1 from Day 7, whereas WS-1 exhibited a delayed response to the increased light intensity (Fig. 3). Eventually, the supplementation of extra light source from Day 6 led to an increase in biomass accumulation in both strains and the final biomass concentration was comparable between M1 and WS-1. With respect to violaxanthin content, both strains reached a maximum level of 1.67 ± 0.27 mg g^− 1^ (WS-1) and 2.10 ± 0.02 mg g^− 1^ (M1) on Day 9. Moreover, in terms of the amount of violaxanthin per unit culture volume, maximal values of 2.25 ± 0.43 mg L^− 1^ (WS-1) and 3.23 ± 0.15 mg L^− 1^ (M1) were observed on Day 10, which corresponded to a 43.54 % increase in the violaxanthin yield in M1 (Fig. 3). These results clearly indicated that the production of violaxanthin from the selected *Nannochloropsis* strains can be reliably performed in a larger cultivation setup and support the possibility of incorporating different light supplementation regimes into the operation of industrial bioreactors, which could in turn further enhance the production of violaxanthin from industrial strains.

### Comparative transcriptomic analysis between WS-1 and M1 strains under normal light condition

To further elucidate transcriptional changes that led to phenotypic differences between WS-1 and M1 under normal light condition, we analyzed differentially expressed genes (DEG) with the raw sequences generated using Illumina Hiseq 2000 platform. Specifically, a total of 18,606 representative transcripts (i.e., unigenes) were identified out of 130 million raw reads; 229 unigenes were eventually identified as differentially expressed genes with LFC greater than 1 (See Additional file 2 for more details). A global survey of the identified DEGs indicated that the number of downregulated DEGs were 3-times more than that of the upregulated DEGs (177 and 52 DEGs, respectively), indicating the downregulation of the metabolic pathways associated with these DEGs as predominant responses in M1 (Additional file 1: Figs. S3 and S4). Subsequent mapping of DEGs to 136 reference KEGG pathway using KO as a reference identified the following 3 KEGG pathways to be enriched with 3 or more DEGs: ko03010 ribosome (10), ko01100 metabolic pathways (9), ko01110 biosynthesis of secondary metabolites (6). Notably, all transcripts associated ribosome were downregulated in M1; combined with the downregulation of Mcm5 [EC: 3.6.4.12] of mini-chromosome maintenance protein (MCM) complex and translation initiation factor 2 subunit 1 (eIF2), these results suggest a delayed protein synthesis and processing in the mutant, attributing to the lower cellular growth rate of M1 under normal light condition [44, 45].

While most of the DEGs enriched in KEGG mapper analysis seemed to be downregulated, the transcripts associated with β-mannosidase [EC: 3.2.1.25], calcium-dependent protein kinase (CPK) [EC: 2.7.11.1], Myosin V (MYO5), selenocysteine insertion sequence-binding protein 2 (SBP2), peptidylprolyl isomerase [EC: 5.2.1.8], and oligopeptidase B (ptrB) [EC: 3.4.21.83] were upregulated in M1, supporting their possible roles in conferring tolerance to high light condition in M1. In particular, CPK is known to play crucial roles in the regulation of plant growth and development and in responses to biotic and abiotic stresses [46]. Moreover, the isomerization of the peptidyl prolyl bonds, a rate-limiting step in protein folding, was upregulated in M1 at the transcription level; such a response was previously reported to play a key role in stress tolerance in cells [47].

Although it could be hypothesized that the transcripts directly associated with the synthesis of violaxanthin and its immediate upstream precursors are also upregulated in M1, DEG analysis did not indicate a substantial upregulation of genes associated with carotenoid synthesis under normal light condition. However, downregulation of violaxanthin de-epoxidase related protein was identified, suggesting that the conversion of violaxanthin to zeaxanthin under high light condition is less active in the thylakoid membrane of M1 [48]. Importantly, according to Jahns et al. [48], the suppression of zeaxanthin formation in plants can lead to an increased damage of photosystems in the short- and long-term [17, 48–50]. However, it should be pointed out that the analysis of DEGs also indicated the upregulation of PsaA. Given that photosystem I (PSI) photoinhibition is related to the degradation of PsaA [51, 52], its upregulation in M1 led us to speculate a greater tolerance of this mutant to PSI photoinhibition especially under high light conditions. Indeed, the cultivation of M1 at HL condition indicated a substantially higher growth of M1 than that of WS-1, and these results seem to suggest that an upregulation of photosystem subunit(s) served as a protective mechanism against damages that might otherwise be expected in photosystems due to reduced zeaxanthin formation in M1.

**Fig. 4:**
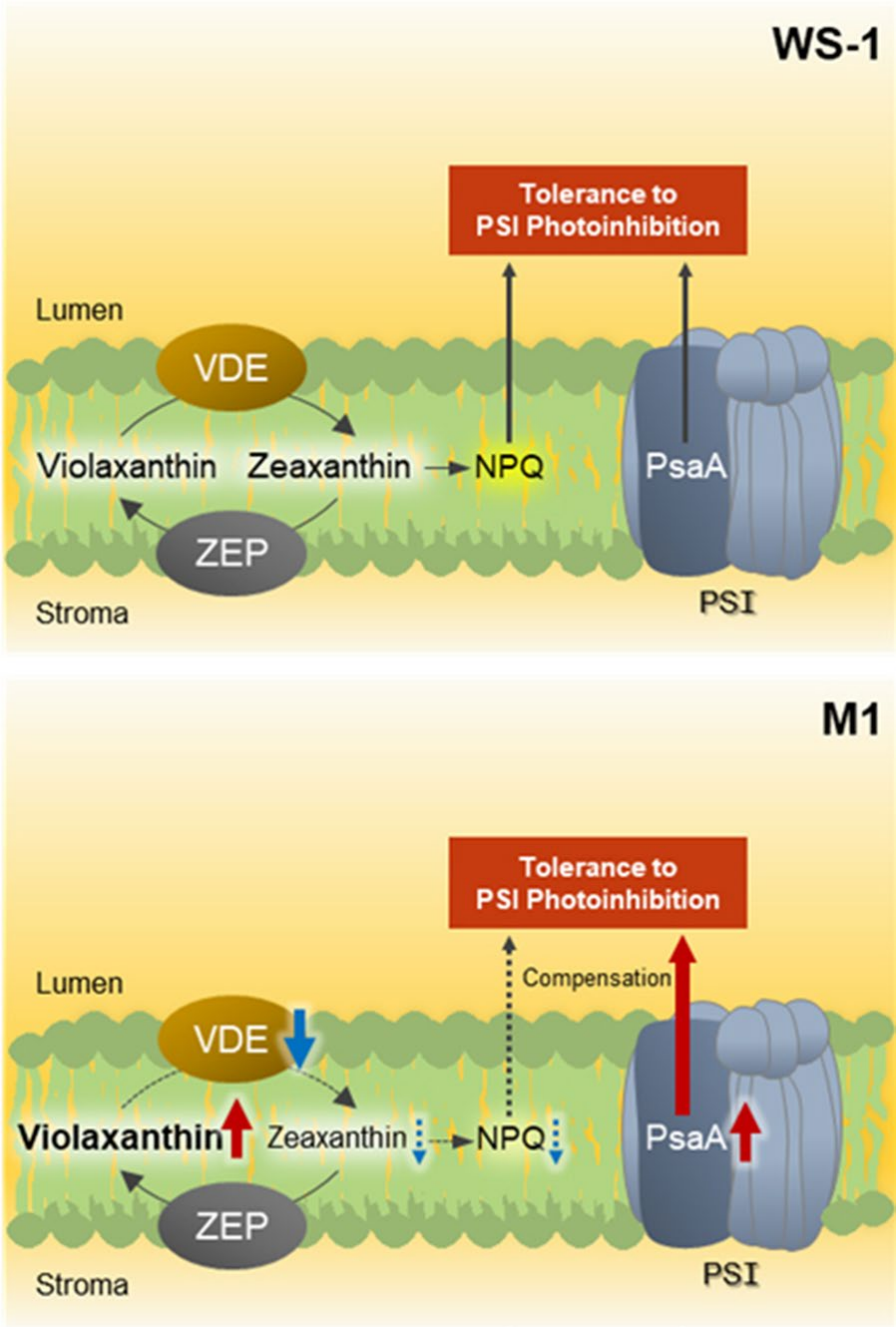
Suggested transcriptional changes between WS-1 and M1 strains. Red and blue arrows indicate up- and down-regulation of transcripts, respectively

To summarize, the results of the comparative transcriptome analysis between WS-1 and M1 identified a relatively small number of DEGs under normal light condition; and these transcripts seemed to play a critical role in maintaining the growth of M1 to the level comparable to WS-1. Furthermore, the results indicated a substantial downregulation of violaxanthin de-epoxidase in the mutant, which could positively contribute to the enhancement of violaxanthin content. Although it is true that other transcripts that were downregulated or the ones that were not differentially expressed under normal light condition could act as key transcriptomic responses at high light stress [48], these results indicated that photoinhibitory damage likely associated with a reduction in the conversion of violaxanthin to zeaxanthin in the mutant is seemingly countered by the upregulation of PsaA (Fig. 4). Further investigations into the responses of *N. oceanica* under different light intensities and growth phases should be performed along with RT-qPCR validation to elucidate key physiological and transcriptional responses associated with the high yield of violaxanthin in M1 under different production scenarios and ultimately develop desirable industrial algal strains; however, DEGs in this study would serve as an important starting point for such endeavor.

## Conclusions

In conclusion, we successfully generated *N. oceanica* mutant (M1) with enhanced violaxanthin content. Compared with the progenitor WS-1, reliably high yield of violaxanthin was confirmed in M1 under different cultivation conditions. Especially, the maximum violaxanthin content (5.21 ± 0.34 mg g^− 1^) obtained during the flask cultivation was one of the highest values reported in the existing literature on microalgae-based production of violaxanthin. The high production of violaxanthin from M1 was further demonstrated in large-scale bioreactor, and the results confirmed the feasibility of violaxanthin production at a scale more relevant to the operation of industrial bioreactor. Comparative transcriptomic analysis suggested key transcriptomic responses that are associated with different physiological changes observed in M1, especially delayed growth under normal light condition and enhanced violaxanthin content. The results also suggested a possible counter mechanism against a putative reduction in photoinhibition tolerance resulted from an increased violaxanthin content and called for further studies on algal responses under different light conditions and growth phases that could provide important insights into maximizing the industrial potential of this promising strain.

## Materials and methods

### Isolation and identification of *Nannochloropsis* stains

Seawater samples were collected from several coastal points of Korean western sea. The samples were spread onto F/2 medium [53] containing 1 % agar with serial dilution suitable for the isolation of individual colonies and incubated at 25 ℃ and normal light intensity (50 µmol photon m^− 2^ s^− 1^). The grown colonies were isolated by micro picking as previously described [54]. Through the followed iterative process, individual algal colonies were isolated and inoculated into the liquid F/2 medium [53] to procure cells for genomic DNA extraction followed by genetic identification. The composition of F/2 medium was (per L): 75 mg of NaNO_3_, 5 mg of NaH_2_PO_4_·H_2_O, 30 mg of Na_2_SiO_3_·9H_2_O, 1 mL of trace metal solution containing 3.15 g of FeCl_3_·6H_2_O, 4.36 g of Na_2_EDTA·2H_2_O, 9.8 mg of CuSO_4_·5H_2_O, 6.3 mg of Na_2_MoO_4_·2H_2_O, 22 mg of ZnSO_4_·7H_2_O, 10 mg of CoCl_2_·6H_2_O and 180 mg of MnCl_2_·4H_2_O per liter and 0.5 mL of vitamin solution containing 200 mg of thiamine HCl, 1 mg of biotin and 1 mg of cyanocobalamin per liter. Partial 18S ribosomal DNA was amplified via PCR using the following primers: forward, 5’ caagtttctgccctatcagct 3’ and reverse, 5’ gctttcgcagtagttcgtctt 3’. Subsequently, the sequence of the PCR product (ca. 1.7 kbp) was analyzed using BLASTn (https://blast.ncbi.nlm.nih.gov/blast.cgi) and 18S rDNA sequence of the strain selected for the subsequent experiments were deposited in NCBI GenBank (Accession #: MW375465).

### Cell cultivations

To isolate *Nannochloropsis* strains, F/2 agar plates were incubated at 25 ℃ under 50 µmol photon m^− 2^ s^− 1^ light intensity. For strain maintenance and all the other experiments, the cells were cultivated in a 50 mL T-flask (SPL, Pocheon, Republic of Korea) with a working volume of 10 mL at 25 ℃ with agitation of 120 rpm. In the experiments for the comparison of light intensity, the intensity of normal light (NL) and high light (HL) were 50 and 150 µmol photon m^− 2^ s^− 1^, respectively, and each intensity condition was confirmed using a LI-250A light meter (LI-COR, NE, USA). Large-scale cultivations were carried out in a 10 L cylindrical bioreactor (custom-made) with 5 L working volume. Air was supplied into the culture solution by using a strong air pump with the flow rate of 25 L min^− 1^ for the aeration and agitation. At the beginning of the cultivation, a flat light source of 300 µmol photon m^− 2^ s^− 1^ was used in only one side of the bioreactor. 6 days after inoculation, another light source of 100 µmol photon m^− 2^ s^− 1^ was introduced in the opposite side to compensate for shading effect.

### Gamma‐ray‐mediated random mutagenesis and mutant screening

*N. oceanica* WS-1 cells at mid-exponential phase were irradiated with gamma-ray in the range of 100 to 1000 Gy using ^60^Co irradiator (Point Source ACEL, IR-79; Nordion Co. Ltd., Ontario, Canada). These experiments were performed at the Korea Atomic Energy Research Institute (Jeong-eup, Republic of Korea). The total activity of ^60^Co source was 80 kCi and the temperature of irradiation room was maintained at 17 ℃. Post gamma-ray irradiation, 10^6^, 10^5^, and 10^4^ cells of each dose were spread onto the F/2 agar plates. Further the cell viability was evaluated by counting the number of colonies. For the mutant screening, 10^8^ of 500 Gy-treated cells were spread onto the F/2 agar plates, and yellowish colonies were picked and inoculated into liquid F/2 medium. During this cultivation, the strains that exhibited good growth were selected for the subsequent experiments.

### Cell counting & calculation of dry cell weight and productivity

To determine the cell growth, volumetric cell numbers were measured using a disposable hemocytometer C-Chips™ (INCYTO, Cheonan, Republic of Korea). A volume of 10 µL culture solution was loaded onto the hemocytometer for counting and the number of cells in the solution was calculated according to the manufacturer’s instructions. Dry cell weight was measured using a pre-dried and pre-weighed GF/C filter paper (Whatman, Maidstone, UK). We filtered 2 mL of culture solution using the filter paper and the culture medium was removed using vacuum pump. It was then rinsed twice with distilled water to remove the residual medium. The filter paper with algal biomass was dried at 105 °C for 24 h and weighed again. The dry cell weight per unit volume was calculated using the following Eq. (1),

1 $${DCW}_{V}=({W}_{F}-{W}_{I})/V$$

where DCW_V_ is dry cell weight per liter (g L^− 1^); W_F_ is the weight of filter paper with algal biomass (g); W_I_ is the weight of empty filter paper (g); and V is the volume of culture solution loaded onto filter paper (L). The biomass productivity was calculated using the following Eq. (2),

2 $$P=({DCW}_{VF}-{DCW}_{VI})/t$$

where P is biomass productivity (g L d^− 1^); F and I represent final and initial points of cultivation, respectively; and t is cultivation time (d). Violaxanthin productivity was calculated using the following Eq. (3),

3 $${P}_{v}=P\times {C}_{v}$$

where P_v_ is violaxanthin productivity (mg L^− 1^ d^− 1^); and C_v_ is violaxanthin content at the final point of cultivation (mg g^− 1^). Here, initial violaxanthin content was assumed to be 0, as it was insignificant.

### High performance liquid chromatography (HPLC)

For the violaxanthin content analysis, total pigments were extracted from wet biomass using ethanol as previously described [55]. In detail, culture solution was spun down after being transferred to 1.5 ml micro centrifuge tube. The wet biomass was then mixed with 1 ml of ethanol and was homogenized via bead beating with zirconia beads (Biospec, OK, USA) for 30 seconds. Subsequently, the tube was subjected to ultrasonication in water for 5 minutes. Finally, the mixture was centrifuged at 12,300×*g* for 1 min. Further, the supernatant was filtered using 0.22 µm PTFE filters and then transferred into an HPLC vial (Whatman). Analysis of pigment content was carried out using Agilent 1260 Infinity Binary LC system (Agilent Tech. Inc., CA, USA) equipped with a Waters Spherisorb® S5 ODS1 4.6 × 250 mm, 5 µm Cartridge Column (Waters Corp., MA, USA). The chromatography was conducted at 40°C for 30 min per sample with an injection volume of 20 µL. The flow rate was set at 1.2 mL min^− 1^ and two mobile phases were used: solvent A comprising 14 % 0.1 M Tris-HCl (pH 8.0), 84 % acetonitrile and 2 % methanol in volume ratio; and solvent B comprising 68 % methanol and 32 % ethyl acetate in volume ratio (solvent gradient: 0 min, 100 % A; 15 min, 100 % A; 19 min, 100 % B (linear); 25 min, 100 % A (linear); 30 min, 100 % A). To identify the peaks observed, retention time of each peak was compared with a series of standard materials including violaxanthin, astaxanthin, lutein, zeaxanthin, chlorophyll *a*, and β-carotene (Sigma Aldrich Co., SL, USA). For quantification, 5, 10, 20, and 50 ppm of violaxanthin standard solutions were used to obtain a calibration curve; the suspected violaxanthin peaks from algal pigment extracts were additionally validated by analyzing a sample spiked with violaxanthin standard. In particular, a spiked sample was prepared by mixing 10 ppm of violaxanthin standard solution with the extracted sample and each of the standard and pigment extract were individually analyzed as well. Thereafter, the retention time and shape of spiked and non-spiked samples were examined.

### Comparative transcriptome analysis between WS-1 and M1 strains

A comparative transcriptome analysis was performed under normal light condition to explore transcription-level differences between wild-type and mutant strains. Of mutants obtained, M1 was selected to be compared to wild-type *N. oceanica* because this mutant exhibited the highest yield of violaxanthin among all tested strains, leading us to expect most distinct transcriptional responses. Both M1 and wild-type strain were grown in triplicated 50 mL T-flasks at normal light condition as described above and the samples reached the stationary phase were collected for transcriptome analysis (Fig. 2a); following the RNA extraction performed as described in Yun et al. [28], library construction and Illumina sequencing of *N. oceanica*, were performed at Seeders Co. (Daejeon, Korea) [28]. Thereafter, de novo assembly was performed based on the previous study; log_2_-fold change (LFC) equal to or greater than 1 was considered for selecting differentially expressed genes (DEGs) with false discovery rate (FDR) lower than 0.01. DEGs were analyzed following the methods previously described [28]; datasets generated from the transcriptome analysis were deposited in NCBI database (Accession #: PRJNA685871).

## Supplementary information

**Additional file 1: Figure S1.** Results from the analysis of three different samples consist of algal extract or violaxanthin standard. Black line (top) indicates the chromatogram of a spiked sample prepared by mixing pigment extract from *Nannochloropsis* sp. WS-1 with violaxanthin standard, green and blue lines (middle and bottom) indicate the chromatograms of the pigment extract from *Nannochloropsis* sp. WS-1 and violaxanthin standard, respectively. Retention times of violaxanthin in each of these sample are 8.968, 8.985, and 8.957; **Figure S2.** HPLC profile of carotenoids and chlorophylls in each of WS-1 and M1. Numbered peaks indicate: (1) unknown; (2) violaxanthin; (3) astaxanthin; (4) lutein; (5) zeaxanthin; (6) chlorophyll *a*; and (7) β-carotene; **Figure S3.** MA plot of comparative transcriptomic analysis. Red and green dots indicate up- and down-regulated DEGs; **Figure S4.** GO plot of comparative transcriptomic analysis; **Table S1.** Viability of gamma-ray-treated cells; **Table S2.** Biomass productivity, violaxanthin content and violaxanthin productivity of WS-1 wild type and 10 mutant strains.

**Additional file 2.** Differential expression gene datasets of the M1 mutant compared to the WS-1 wild-type.

## Data Availability

All data generated or analysed during this study are included in this published article.
