# Supplementary material for: Development of a novel nannochloropsis strain with enhanced violaxanthin yield for large‐scale production
Source: Microb Cell Fact. 2021 Feb 15;20:43. doi: 10.1186/s12934-021-01535-0 (PMC7885382; doi:10.1186/s12934-021-01535-0)
Supplement: Supplementary file 1 — Additional file 1: Figure S1. Results from the analysis of three different samples consist of algal extract or violaxanthin standard. Black line (top) indicates the chromatogram of a spiked sample prepared by mixing pigment extract from Nannochloropsis sp. WS-1 with violaxanthin standard, green and blue lines (middle and bottom) indicate the chromatograms of the pigment extract from Nannochloropsis sp. WS-1 and violaxanthin standard, respectively. Retention times of violaxanthin in each of these sample are 8.968, 8.985, and 8.957; Figure S2. HPLC profile of carotenoids and chlorophylls in each of WS-1 and M1. Numbered peaks indicate: (1) unknown; (2) violaxanthin; (3) astaxanthin; (4) lutein; (5) zeaxanthin; (6) chlorophyll a; and (7) β-carotene; Figure S3. MA plot of comparative transcriptomic analysis. Red and green dots indicate up- and down-regulated DEGs; Figure S4. GO plot of comparative transcriptomic analysis; Table S1. Viability of gamma-ray-treated cells; Table S2. Biomass productivity, violaxanthin content and violaxanthin productivity of WS-1 wild type and 10 mutant strains. [file 12934_2021_1535_MOESM1_ESM.docx]

**Development of a novel *Nannochloropsis* strain with enhanced violaxanthin yield for large-scale production**

Su-Bin Park^1,2,†^, Jin-Ho Yun^1,†^, Ae Jin Ryu^1^, Joohyun Yun^1^, Ji Won Kim^1,2^, Sujin Lee^1,2^, Saehae Choi^1,3^, Dae-Hyun Cho^1^, Dong-Yun Choi^1^, Yong Jae Lee^1,2*^, Hee-Sik Kim^1,2*^

^1^Cell Factory Research Center, Korea Research Institute of Bioscience and Biotechnology (KRIBB), Daejeon 34141, Republic of Korea

^2^Major of Environmental Biotechnology, KRIBB School of Biotechnology, Korea University of Science and Technology (UST), Daejeon 34113, Republic of Korea

^3^Osong Medical Innovation Foundation, Chungbuk 28160, Republic of Korea

^*^Correspondence: leeyj@kribb.re.kr; hkim@kribb.re.kr

^†^Su-Bin Park and Jin-Ho Yun contributed equally to this work

**
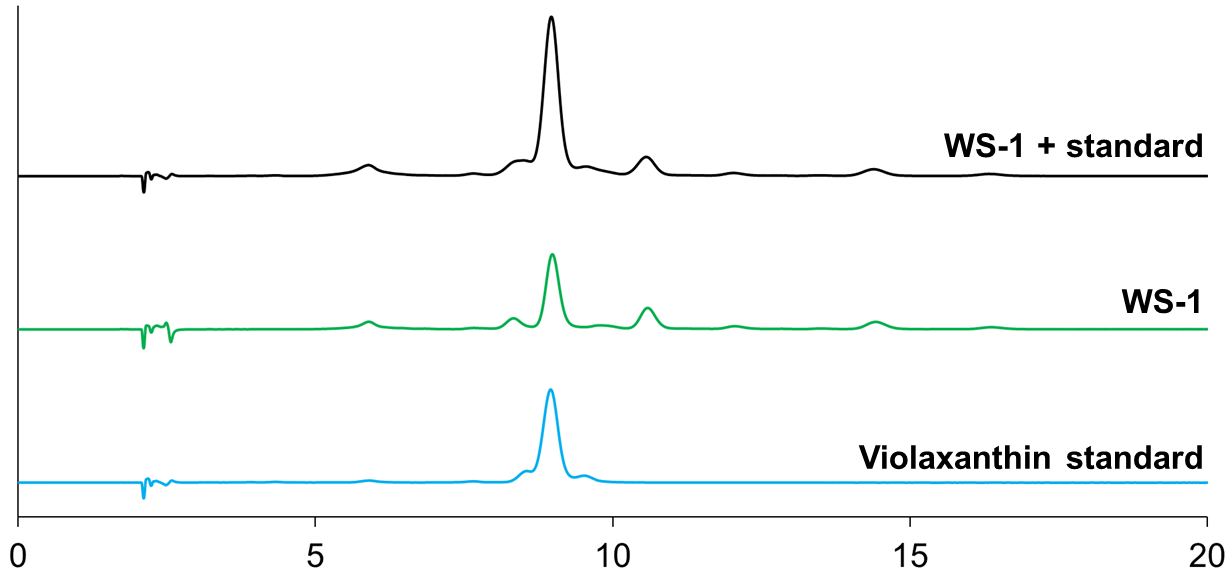
**

**Figure S1.** Results from the analysis of three different samples consist of algal extract or violaxanthin standard. Black line (top) indicates the chromatogram of a spiked sample prepared by mixing pigment extract from *Nannochloropsis* sp. WS-1 with violaxanthin standard, green and blue lines (middle and bottom) indicate the chromatograms of the pigment extract from *Nannochloropsis* sp. WS-1 and violaxanthin standard, respectively. Retention times of violaxanthin in each of these sample are 8.968, 8.985, and 8.957.

**
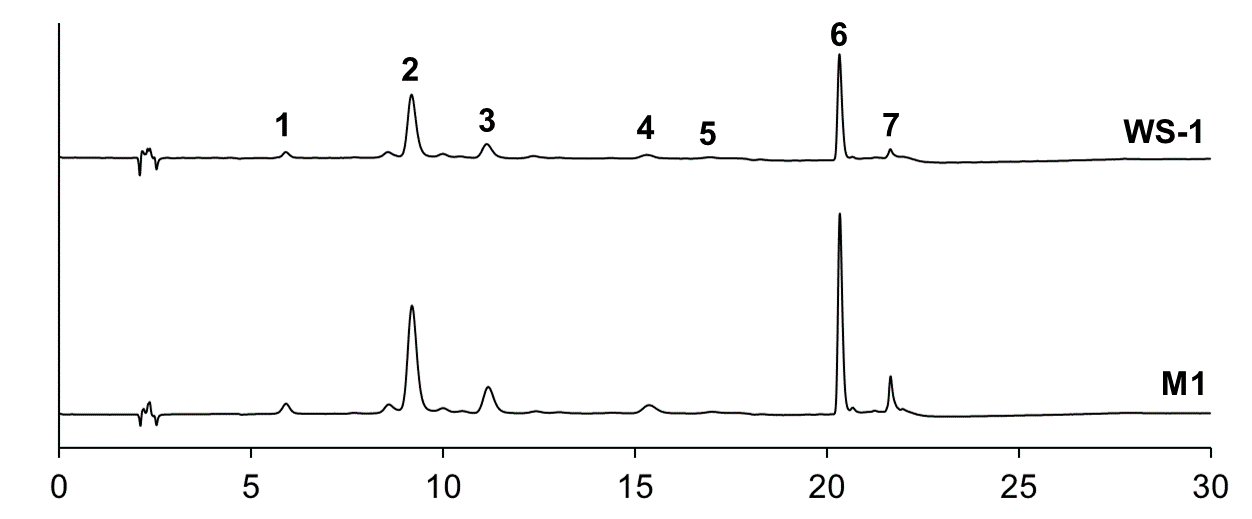
**

**Figure S2.** HPLC profile of carotenoids and chlorophylls in each of WS-1 and M1. Numbered peaks indicate: (1) unknown; (2) violaxanthin; (3) astaxanthin; (4) lutein; (5) zeaxanthin; (6) chlorophyll *a*; and (7) β-carotene.

**
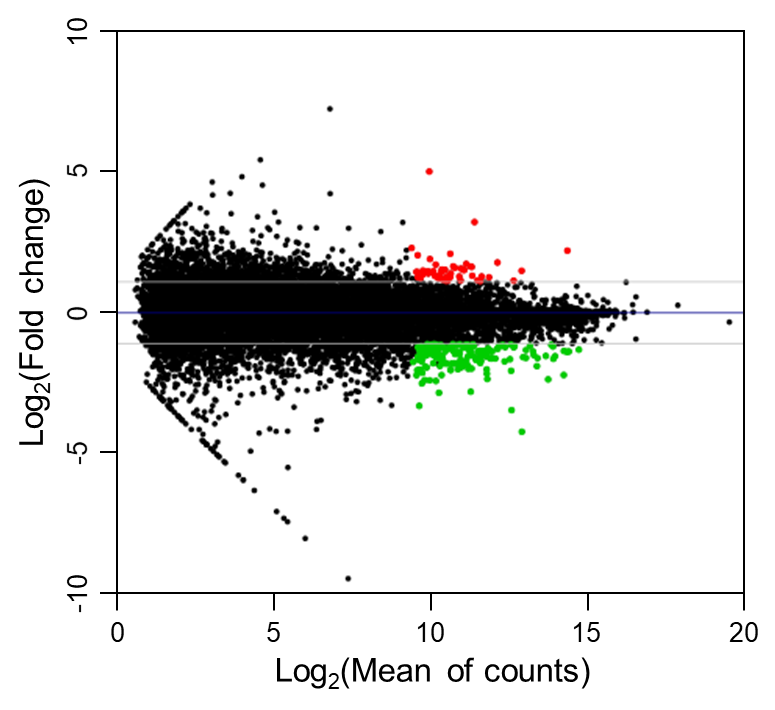
**

**Figure S3.** MA plot of comparative transcriptomic analysis. Red and green dots indicate up- and down-regulated DEGs.

**
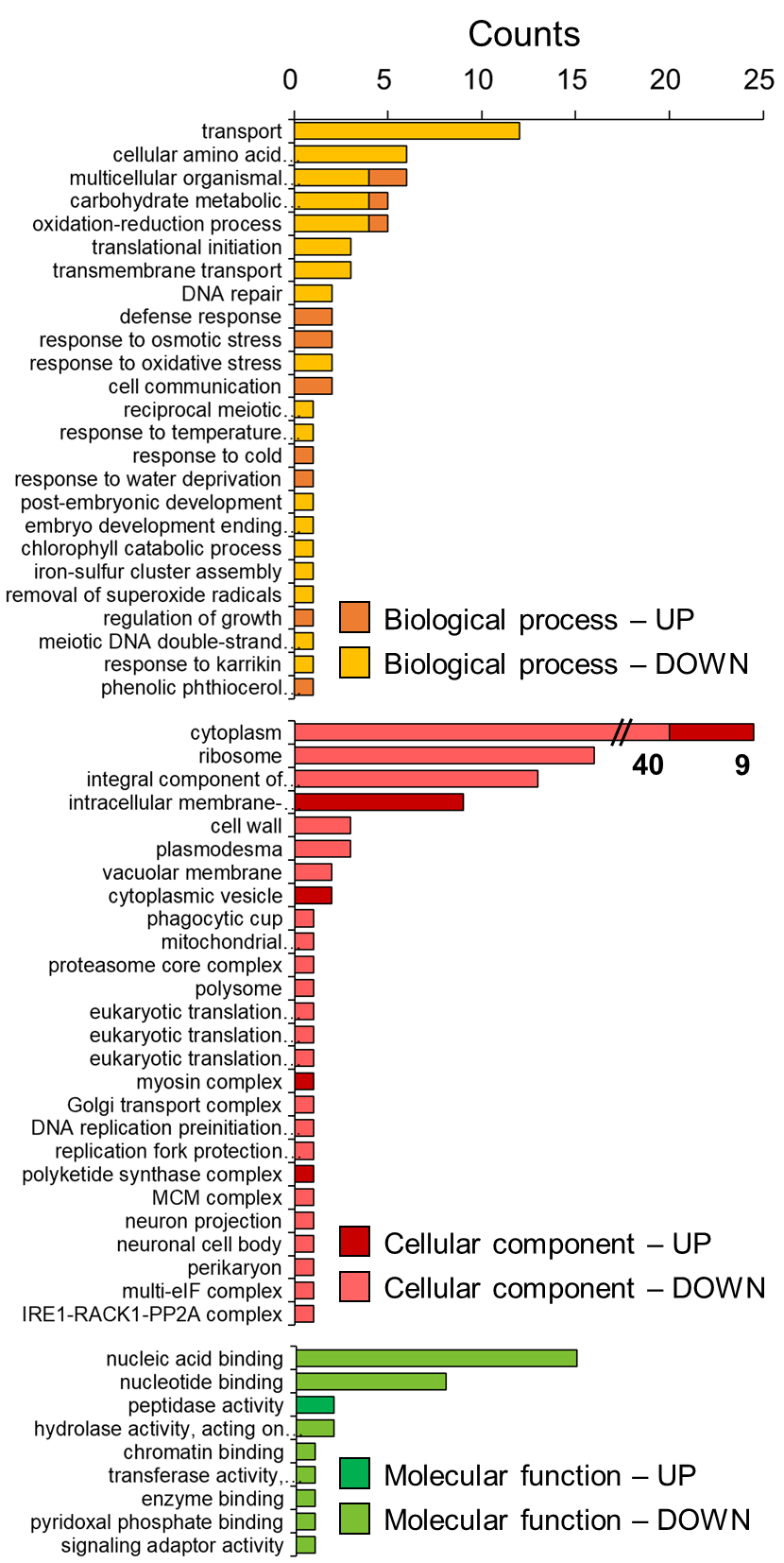
**

**Figure S4.** GO plot of comparative transcriptomic analysis.

**Table S1.** Viability of gamma-ray-treated cells.

| Gamma-ray (Gy) | Viability (%) |
| --- | --- |
| 100 | 16.000 |
| 200 | 3.000 |
| 300 | 0.500 |
| 400 | 0.300 |
| 500 | 0.015 |
| 700 | 0.000 |
| 1000 | 0.000 |

**Table S2.** Biomass productivity, violaxanthin content and violaxanthin productivity of WS-1 wild type and 10 mutant strains.

| Strains | Final cell density  (g L^-1^) | Biomass productivity  (g L^-1^ d^-1^) | Violaxanthin content  (mg g^-1^) | Violaxanthin productivity  (mg L^-1^ d^-1^) |
| --- | --- | --- | --- | --- |
| WS-1 | 2.4937 | 0.2078 | 3.4172 | 0.7101 |
| M1 | 2.5217 | 0.2101 | 4.0874 | 0.8589 |
| M2 | 2.3263 | 0.1662 | 3.2848 | 0.5458 |
| M3 | 1.9354 | 0.1613 | 4.3096 | 0.6951 |
| M4 | 2.1681 | 0.1807 | 4.1858 | 0.7563 |
| M5 | 2.6985 | 0.2249 | 2.6227 | 0.5898 |
| M6 | 2.8535 | 0.1783 | 2.4183 | 0.4313 |
| M7 | 1.8998 | 0.1357 | 2.5320 | 0.3436 |
| M8 | 1.0701 | 0.1070 | 3.3327 | 0.3566 |
| M9 | 2.5821 | 0.1844 | 0.4552 | 0.0840 |
| M10 | 2.0161 | 0.1440 | 1.9803 | 0.2852 |
